# The Role of Landiolol in Coronary Artery Disease: Insights into Acute Coronary Syndromes, Stable Coronary Artery Disease and Computed Tomography Coronary Angiography

**DOI:** 10.3390/jcm14155216

**Published:** 2025-07-23

**Authors:** Athina Nasoufidou, Marios G. Bantidos, Panagiotis Stachteas, Dimitrios V. Moysidis, Andreas Mitsis, Barbara Fyntanidou, Konstantinos Kouskouras, Efstratios Karagiannidis, Theodoros Karamitsos, George Kassimis, Nikolaos Fragakis

**Affiliations:** 1 Second Cardiology Department, Medical School, Hippokration General Hospital, Aristotle University of Thessaloniki, 54124 Thessaloniki, Greece; mbadidos@gmail.com (M.G.B.); nfrag@auth.gr (N.F.); 2 Faculty of Health Sciences, School of Medicine, Aristotle University of Thessaloniki, 54124 Thessaloniki, Greece; 3 Cardiology Department, Nicosia General Hospital, State Health Services Organization, Nicosia 2029, Cyprus; andymits7@gmail.com; 4 Department of Emergency Medicine, AHEPA University Hospital, 54636 Thessaloniki, Greece; bfyntan@yahoo.com (B.F.); stratoskarag@gmail.com (E.K.); 5 Department of Radiology, AHEPA University General Hospital, Aristotle University of Thessaloniki, 54124 Thessaloniki, Greece; 6 First Department of Cardiology, AHEPA Hospital, Aristotle University of Thessaloniki, 54124 Thessaloniki, Greece

**Keywords:** landiolol, coronary syndrome, computed tomography coronary angiography, coronary artery disease

## Abstract

Coronary artery disease (CAD) constitutes a major contributor to morbidity, mortality and healthcare burden worldwide. Recent innovations in imaging modalities, pharmaceuticals and interventional techniques have revolutionized diagnostic and treatment options, necessitating the reevaluation of established drug protocols or the consideration of newer alternatives. The utilization of beta blockers (BBs) in the setting of acute myocardial infarction (AMI), shifting from the pre-reperfusion to the thrombolytic and finally the primary percutaneous coronary intervention (pPCI) era, has become increasingly more selective and contentious. Nonetheless, the extent of myocardial necrosis remains a key predictor of outcomes in this patient population, with large trials establishing the beneficial use of beta blockers. Computed tomography coronary angiography (CTCA) has emerged as a highly effective diagnostic tool for delineating the coronary anatomy and atheromatous plaque characteristics, with the added capability of MESH-3D model generation. Induction and preservation of a low heart rate (HR), regardless of the underlying sequence, is of critical importance for high-quality results. Landiolol is an intravenous beta blocker with an ultra-short duration of action (t1/2 = 4 min) and remarkable β1-receptor specificity (β1/β2 = 255) and pharmacokinetics that support its potential for systematic integration into clinical practice. It has been increasingly recognized for its importance in both acute (primarily studied in STEMI and, to a lesser extent, NSTEMI pPCI) and chronic (mainly studied in elective PCI) CAD settings. Given the limited literature focusing specifically on landiolol, the aim of this narrative review is to examine its pharmacological properties and evaluate its current and future role in enhancing both diagnostic imaging quality and therapeutic outcomes in patients with CAD.

## 1. Introduction

Coronary artery disease (CAD) is a main leading cause of morbidity and mortality worldwide. CAD is also a major contributor to disability, negatively affecting patients’ quality of life and functional capacity [1].

Acute coronary syndromes (ACSs) constitute the primary clinical manifestation of CAD. During an ACS event, the heart strives to maintain a balance between myocardial oxygen demand and the coronary blood supply. Beta blockers (BBs) play a vital role in improving coronary reperfusion and exhibiting anti-arrhythmic properties, making them an effective cardioprotective medication when used in appropriate dosage [2].

Furthermore, computed tomography coronary angiography (CTCA) is a contemporary diagnostic tool for the visualization of the extent of CAD and the description of the plaque characteristics. For the best image quality in CTCA, it is essential to maintain a low HR, usually around 60 beats per minute (bpm), which is often not feasible without the use of antiarrhythmic drugs, particularly BBs. Intravenous BBs can rapidly achieve the targeted HR, contributing to better image quality and lower radiation doses [3].

**Figure 1:**
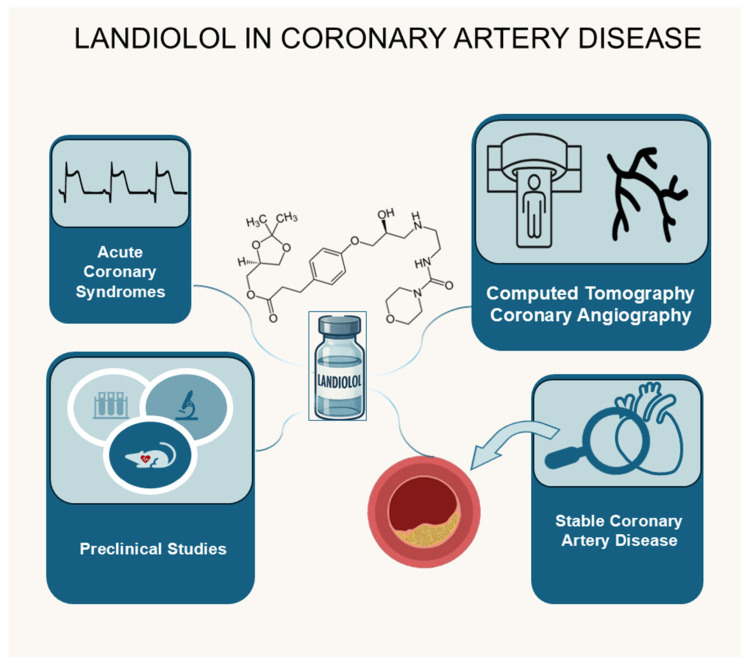
Landiolol in coronary artery disease.

This review is the first to consolidate and critically appraise all available evidence on landiolol across the full spectrum of CAD, including acute coronary syndromes, CTCA imaging, stable angina, and experimental animal studies. No prior publication has integrated these diverse data sources into a focused analysis centered exclusively on this underutilized drug. By comprehensively detailing landiolol’s pharmacologic profile and consistently favorable effects, this synthesis not only underscores its unique therapeutic potential but also challenges existing practice paradigms and offers a fresh framework to guide future research and support broader clinical adoption. Figure 1 presents an overview of the uses of landiolol in CAD.

### 1.1. BBs in ACS Recommendations

Intravenous BBs are recommended in the acute management of ST-elevation myocardial infarction (STEMI) based on European Society of Cardiology (ESC) guidelines (Class IIa recommendation, Level of Evidence A). Among these, metoprolol is the preferred agent due to its extensive evaluation in clinical studies. Beta-blockers reduce myocardial oxygen demand by lowering heart rate (HR,) facilitating coronary reperfusion [4].

Additionally, BBs are essential for managing arrhythmias associated with acute coronary syndromes (ACSs). They are indicated for heart rate control in supraventricular tachyarrhythmias (SVTs), most commonly atrial fibrillation (AF) (Class I recommendation, Level of Evidence C), and for ventricular arrhythmias complicating ACSs (Class I recommendation, Level of Evidence B) [4].

Landiolol, a beta-blocker with favorable pharmacokinetics, may provide additional advantages in this context. Nevertheless, the use of intravenous BBs in non-ST-elevation myocardial infarction (NSTEMI) or unstable angina remains insufficiently studied.

### 1.2. Landiolol Properties

Landiolol, an intravenous beta-blocker, possesses remarkable pharmacological properties, making it highly effective in various clinical scenarios. It has an exceptionally high specificity for beta-1 adrenergic receptors (β1/β2 = 255) and a very short half-life of approximately four minutes, allowing for a rapid onset and offset of action [5]. Due to this selectivity, landiolol significantly reduces HR with only a minimal decrease in blood pressure (BP), making it a preferred choice in hemodynamically unstable patients presenting with tachycardia. In the event of hypotension, its short half-life allows for rapid hemodynamic recovery upon discontinuation of the infusion, minimizing the risk of prolonged period of low BP and associated complications. Additionally, landiolol can be titrated every 10 min if HR control remains insufficient. Its dosing is tailored to the patient’s cardiac function and body weight, ensuring a personalized and effective approach to treatment [6].

Landiolol and esmolol are both BBs that share similarities in the metabolism pathway. However, landiolol demonstrates faster pharmacokinetics, greater cardioselectivity and reduced negative inotropic effect, as clinical studies have shown [6]. In Table 1 we provide a summary of landiolol properties.

## 2. Landiolol in Acute Coronary Syndromes (ACSs): Clinical Evidence and Study Outcomes

### 2.1. Reduction in Myocardial Salvage Index (MSI)

MSI is defined as the difference between the area at risk and the area of necrosis assessed in cardiac magnetic resonance (CMR) studies. In the latest meta-analysis about intravenous BBs in STEMI patients, MSI was improved when BBs were used early before reperfusion. This benefit was more pronounced in cases involving lesions in the left anterior descending artery [7]. This meta-analysis included three studies on landiolol, two on esmolol and two on metoprolol. Among these, the key study by Miyamoto demonstrated that landiolol reduced MSI in CMR performed 5–7 days post-reperfusion in STEMI patients with Killip I/II classification, compared to placebo [8,9].

### 2.2. Reduction in Myocardial Oxygen Demand Hemodynamic Effects

Landiolol provides rapid HR reduction without significant hypotension, a particularly important aspect in ACS patients. In a Japanese study that included patients with AMI and unstable angina, landiolol effectively reduced HR without lowering systolic or diastolic BP [10]. The Kiyokuni study reported a lower progression to Killip Class III or IV with landiolol compared to placebo (0% vs. 10%, *p* = 0.028), indicating a beneficial hemodynamic effect [11]. Multiple RCTs found no significant differences in systolic or diastolic BP between landiolol and placebo [8,12], while an observational study reported a lower incidence of hypotension with landiolol compared to placebo (15% vs. 32%, *p* = 0.046) [11]. Additionally, myocardial infarction damage, as measured by peak creatine kinase (CK) and CK area under the curve (CK-AUC), was significantly reduced in the landiolol group among anterior STEMI patients (3107.0 ± 1575.1 vs. 5078.2 ± 2748.2 U/L, *p* = 0.0394) [13].

### 2.3. Percutaneous Coronary Intervention Results

Optimal reperfusion has been also assessed in STEMI patients treated with landiolol. ST-segment resolution (STR: 64% vs. 42%, *p* = 0.023) and myocardial blush grade (MBG: 64% vs. 45%, *p* = 0.045) was achieved in higher rates compared to placebo [11].

### 2.4. Arrhythmia Management

In the clinical setting of STEMI, the risk of fatal arrhythmias, such as ventricular fibrillation (VF) and ventricular tachycardia (VT), is high. Furthermore, non-sustained ventricular tachycardia (NSVT), although not directly life-threatening, may pose an additional risk of destabilizing cardiac rhythm. An observational Japanese study comparing landiolol to placebo reported a lower incidence of NSVT (27% vs. 50%, *p* = 0.014) [11]. Nevertheless, a recent subgroup analysis of RCTs found no significant difference in the incidence of VT/VF between landiolol and placebo groups [7].

### 2.5. Safety Outcomes in ACSs

Safety outcomes are a critical consideration when administering intravenous beta-blockers in emergency settings. A 2019 meta-analysis, which included four RCTs, evaluated the incidence of death or myocardial infarction, as a primary outcome, within one year. The analysis found no significant difference between beta-blockers and placebo, supporting the safety profile of intravenous BBs in this context [14]. Among the four studies, one was focused on landiolol [12]. Bradycardia is a potential side effect of landiolol administration but is typically sinus bradycardia, which often resolves upon discontinuation of the drug without requiring further intervention [10,12]. The rates of serious adverse events such as cardiogenic shock, atrioventricular block or bradycardia requiring pharmacologic intervention or pacemaker implantation were not significantly different between landiolol and control groups [7,8,13].

### 2.6. Long Term Outcomes

Additionally, to their critical role in the acute phase of STEMI management, intravenous BBs have also been extensively studied regarding their long-term effects. In the Kiyokuni observational study with a 12-month follow-up, the incidence of heart failure requiring hospitalization was lower in the landiolol group. However, rates of cardiac death, non-fatal myocardial infarction, non-fatal stroke and target vessel revascularization did not differ significantly between groups [11]. Similarly, a sub-analysis of an observational study reported a lower rate of worsening heart failure at six months in the landiolol group, though the difference did not reach statistical significance (0% vs. 7%, *p* = 0.07) [15]. However, a meta-analysis found no significant difference in heart failure readmission rates [7].

B-type natriuretic peptide (BNP) levels, a biomarker for heart failure severity, were significantly lower in the landiolol group at six months in one RCT (63.1 ± 56.3 pg/mL vs. 40.0 ± 27.3 pg/mL, *p* < 0.05) [16], though another study found no significant difference [17].

Three RCTs evaluated left ventricular ejection fraction (LVEF) outcomes. One reported improved LVEF in the landiolol group at six months, though the difference was not statistically significant [16]. Another study found a statistically significant improvement in LVEF at six months (52.0 ± 1.5% vs. 49.1 ± 1.5%, *p* = 0.01) [12]. A third study reported significant LVEF improvements measured by both ultrasound (UCG: 3.06 ± 8.26% vs. 8.21 ± 10.91%, *p* = 0.04) and Tc-MIBI scintigraphy (8.83 ± 7.44% vs. 3.35 ± 10.11%, *p* = 0.04) [17].

Changes in left ventricular end-diastolic volume (LVEDV) and left ventricular end-systolic volume (LVESV) were also examined in the chronic phase. Hanada et al. reported a significant increase in LVEDV in the control group (78.0 ± 2.7 vs. 72.5 ± 2.8 mL/m^2^, *p* = 0.02) [12], and Fugita demonstrated that LVEDV was better in the landiolol group and also statistically significant (89.9 ± 27.1 mL vs. 113.2 ± 48.6 mL, *p* = 0.04) [17]. Both studies reported no difference in LVESV index or LVESV, respectively. These changes reflect the chronic changes in the EF in the aforementioned studies.

A summary of these findings is provided in Table 2.

## 3. Landiolol in Computed Tomography Coronary Angiography (CTCA)

CTCA is a non-invasive imaging modality widely used for the assessment of coronary artery anatomy and detection of CAD. Optimal image quality requires a low and stable heart rate, typically below 60 bpm, to minimize motion artifacts. Achieving this target heart rate is often challenging and time-consuming. Landiolol is known to achieve rapid HR reduction with a favorable safety profile. Dosing and administration are also important factors, since landiolol can be titrated easily through dosing charts and easily ceased if needed. So, incorporating landiolol into CTCA protocols can enhance diagnostic efficiency and patient comfort [18,19].

### 3.1. Heart Rate Reduction and Normalization Timeline

Multiple studies have shown that landiolol produces a marked reduction in HR [20,21,22,23,24,25,26,27], with a significant effect observed as early as 5 to 15 min after administration (at doses of 0.06 mg/kg and 0.125 mg/kg [20], and sustained reductions persisting for at least 30 min compared to controls) [20,21]. In an RCT employing different BBs, the HR was significantly lower in the propranolol group compared to the low-dose (bolus dose of 0.125 mg/kg) landiolol group. Also, while HR tended to be lower in the high-dose (bolus dose of 0.125 mg/kg +3.75 mg/kg) compared to the low-dose landiolol group, the findings were of no statistical significance (*p* = 0.10) [28]. A clear trend of HR reduction dose-dependency was established in a different observational study (higher doses achieving greater reductions), where an initial dose of 0.125 mg/kg was doubled and quadrupled (*p* = 0.0008 and 0.0109, respectively) [22]. Furthermore, the stability (decrease in variability) of HR during CT acquisition following landiolol administration has been evaluated and found superior to the pre-administration state (*p* < 0.05) [23].

### 3.2. Image Quality Analysis

Across multiple studies evaluating landiolol in CTCA, improvements in image quality have been consistently reported. In a multicenter RCT, landiolol administration was associated with a significantly higher proportion of correctly classified cases per patient, per artery, and per segment, while the number of assessable coronary segments also increased, particularly at doses of 0.125 mg/kg [20]. In other studies, image quality, assessed both at optimal and mid-diastolic reconstruction phases, was significantly better in the landiolol groups compared to controls, receiving higher scores (2 or 3) across subjects, vessels, and segments (*p* < 0.0001) [21]. Dose-dependent effects were observed in one trial of different BBs, where no significant difference in image quality was found between propranolol and low-dose (bolus dose of 0.125 mg/kg) landiolol groups (*p* = 0.91). Although, high-dose (bolus dose + 3.75 mg) landiolol resulted in improved image quality compared to the low-dose group (*p* = 0.02) [28]. In a multicenter observational study, coronary stenosis was diagnosable in all groups, with no significant difference in interpretability reported [22]. Finally, one study investigated the reduction in radiation dose associated with landiolol use, with a nearly 50% decrease in mean exposure compared to pre-administration levels (*p* < 0.001) [24].

### 3.3. Safety Outcomes in CTCA

Landiolol’s safety has mostly been assessed through BP variation and adverse events requiring or not cessation of drug administration. There was either no statistically significant difference in BP reduction between the control and landiolol groups [22,23,27], or when a difference was observed, it was no longer significant at the 30 min time point [20,21]. In a study that monitored the ejection fraction (EF), the results indicated no statistically significant changes [25]. Most studies reported no adverse events [23,24,26,27], or no difference in adverse events between the control and landiolol groups [20]. In a RCT using a single bolus dose of 0.125 mg/kg, there were two notable adverse events reported: one patient experienced bradycardia and another a decrease in BP. They were both asymptomatic and recovered within 5 min and 10 min, respectively [28]. Moreover, in an observational study utilizing a single bolus injection of 16.1 ± 7.4 mg, eight patients (4.5% of total) displayed symptoms (two floating feeling, two nauseous, one vomiting, one feeling bad, one hypotensive and one lightheaded) [29]. Overall, no serious adverse events were observed requiring discontinuation or additional medical intervention in any of studies reviewed.

A summary of these findings is provided in Table 3.

## 4. Landiolol in Stable CAD: Clinical Evidence and Study Outcomes

Potential benefits of landiolol use in the setting of interventional treatment of stable CAD have been inspected to a much lesser extent. Few RCTs have investigated its effects on patients undergoing elective PCI for stable angina or asymptomatic coronary lesions.

In an RCT implementing brief intracoronary landiolol administration (0.06 mg/kg) in patients undergoing elective PCI, both incidences of postprocedural myocardial injury and MI (cTnI elevation ≥0.04 ng/mL and ≥0.11 ng/mL, respectively) were significantly lower [30]. In subsequent research, the same author demonstrated that a combination of intracoronary and intravenous landiolol administration—specifically, 0.06 mg/kg given before and after balloon inflation or a six-hour intravenous infusion at 0.02 mg/kg/min—resulted in similarly significant cardioprotective effects compared to no treatment [31]. The incidence of myocardial injury dropped from 79% to 56% (*p* = 0.044), and myocardial infarction from 70% to 41% (*p* = 0.016). However, it is noteworthy that the left ventricular wall motion score index (LVWSI), a semi-quantitative measure of LVEF, did not show any significant improvement 14–30 days post-PCI in either group. This raises questions about the true clinical relevance of these findings.

In another study focusing on stable angina patients undergoing elective PCI, intravenous landiolol administration did not differ from the control group in three-day post-PCI levels of adiponectin (APN) and high-molecular-weight APN, molecules thought to correlate with cardioprotection (e.g., bisoprolol has been shown to increase them, reducing myocardial damage) [32]. The absolute change in high-molecular-weight APN levels was significantly smaller in the landiolol group one day after PCI (*p* = 0.031), although there was still a significant reduction from initial levels (*p*  <  0.001). Thus, it needs to be clarified by further studies whether this mitigation of decline is of clinically measurable benefit.

Regarding safety, no incidences of coronary spasm, bradycardia, or congestive heart failure were reported in any of the patients—a finding that may reflect the drug’s favorable pharmacokinetic and pharmacodynamic properties, along with close clinical monitoring and a well-controlled treatment protocol.

## 5. Landiolol in Animal Studies with CAD

Data are available on electrophysiological properties, oxygen-consumption and hemodynamic parameters. Landiolol was found to have no effect on cardiomyocyte resting and action potential amplitude and minimal effect on action potential duration in a study of mechanically perfused excised guinea pig hearts, while HR reduction was modest and dose-dependent [33]. Regarding ischemia-reperfusion injury (IRI), landiolol was shown to have similar cardioprotective effects to ischemic precondition (IPC) enhancing coronary flow, myocardial oxygen consumption, and reducing infarct size after long episodes of global ischemia, but no synergic action with IPC was observed in isolated rat hearts [34]. Another study assessing response to IRI demonstrated possible anti-ischemic and antioxidative effects of landiolol, since levels of tissue malondialdehyde (a highly reactive oxidative enol) were markedly lower in the group of guinea pigs, where the drug was provided compared to control [35]. Finally, when administered intracoronary in induced IRI settings in living pigs, landiolol seemed to avert segmental wall thickening, especially of the anterior wall (provoked by left anterior descending occlusion and reperfusion), and decrease left ventricular systolic pressure. In a second series of these experiments, no significant changes on HR and cardiac output were noticed, while cardioprotection was illustrated again, this time via lower CK-MB levels and less sub-sarcolemmal blebbing (a sign of irreversible ischemic injury) [36].

## 6. Discussion

Landiolol is a highly selective, ultra-short-acting β1-blocker that offers significant advantages over conventional beta-blockers. Its rapid onset, short half-life and minimal impact on BP make it particularly advantageous in clinical settings requiring precise heart rate control, especially when BP is borderline or unstable. ACS is a situation where heart rate control is essential to reduce myocardial oxygen demand and limit ischemic injury. The ESC guidelines recommend the use of intravenous BBs during the acute phase of STEMI, with metoprolol being the most extensively studied agent (Class IIa, Level A) [4]. Metoprolol has demonstrated not only hemodynamic benefits through heart rate reduction but also potential anti-inflammatory and cardioprotective effects. Landiolol emerges as a valuable alternative, particularly in patients at higher risk of hemodynamic compromise, due to its ultra-short-acting profile and high β1-selectivity. Ongoing studies are exploring novel therapeutic targets in ACS to improve patient outcomes and reduce mortality [37]. Among these, anti-inflammatory agents have emerged as a promising area of interest, given the central role of inflammation in the pathophysiology of ACS [38]. Landiolol has shown efficacy in the management of both supraventricular arrhythmias, such as atrial fibrillation, and ventricular arrhythmias complicating ACS. These arrhythmias are associated with increased morbidity and mortality and require prompt and safe rate control. Notably, the study by Kiyokuni et al. demonstrated that landiolol significantly reduced progression to higher Killip classes (III/IV) compared to placebo (0% vs. 10%, *p* = 0.028), indicating better hemodynamic stability and a reduced incidence of acute heart failure [32]. This is particularly important, given that heart failure following ACS often leads to recurrent hospitalizations and long-term mortality. Our recent meta-analysis assessing the use of landiolol in patients with supraventricular arrhythmias and left ventricular dysfunction further supports its efficacy and safety [39]. These patients typically present with lower baseline blood pressures due to impaired cardiac output, highlighting the need for agents with minimal hypotensive effects. Multiple RCTs have consistently shown that landiolol does not significantly lower systolic or diastolic BP when compared to placebo, underlining its hemodynamic tolerability [9,12,16]. Observational data also reveal a lower incidence of hypotension in patients treated with landiolol versus placebo, emphasizing the importance of individualized hemodynamic assessment and careful monitoring in the acute setting [10,11]. Additionally, landiolol has demonstrated a cardioprotective effect in anterior STEMI. In one study, patients receiving landiolol had significantly smaller infarct sizes, as indicated by lower peak CK levels and total CK-AUC compared to those receiving placebo (3107.0 ± 1575.1 vs. 5078.2 ± 2748.2 U/L, *p* = 0.0394) [13]. This suggests that beyond heart rate control, landiolol may contribute to limiting myocardial damage during acute ischemia.

HR reduction and stabilization are essential for obtaining high-quality images during CTCA. Compared to oral beta-blockers, landiolol offers a more rapid onset of action and highly predictable pharmacodynamics, allowing clinicians to titrate the dose in real time to achieve optimal HR control. Its ultra-short half-life minimizes the risk of prolonged negative inotropic effects, providing a significant advantage in patients who may be hemodynamically vulnerable. Studies have demonstrated that the use of landiolol leads to improved image quality, greater diagnostic accuracy and lower radiation dosage [40], particularly in patients presenting with elevated baseline heart rates at the start of CTCA [21]. In addition to its efficacy, landiolol has shown a favorable safety profile in this setting, with minimal negative inotropic effects. In most cases, no additional intervention is needed beyond adjusting or discontinuing the infusion if adverse effects occur [29]. This makes landiolol a safe and effective option for HR control in both outpatient imaging and emergency diagnostic scenarios.

An important consideration during CTCA is the potential for respiratory side effects associated with β-blocker use. In a comparative study of five β-blockers, landiolol, propranolol and atenolol achieved the target HR reduction (ΔHR > 10%) at the initiation of CTCA, unlike atenolol and propranolol. However, landiolol demonstrated the most favorable profile, with a ΔHR of 13.5% at the start and near-complete resolution by the end of the scan (0.3%). Critically, landiolol was associated with the lowest respiratory impact, with a change in Forced Expiratory Volume in 1 s (FEV1) of just 0.04–2.5%, highlighting its optimal balance of efficacy and safety for heart rate control during CTCA procedures [41].

An additional key consideration is the cost-effectiveness of landiolol, especially given its status as a prototype drug. In a cost-minimization analysis conducted in Japan, researchers evaluated the use of landiolol in tachycardic patients undergoing coronary CT angiography (CTCA) for suspected coronary artery disease. Using a decision-tree model, the study compared costs associated with landiolol versus placebo. Landiolol significantly improved the CTCA success rate (81.4% vs. 54.2%), thereby reducing the need for additional, more invasive and costly procedures, such as coronary angiography. As a result, the expected cost per patient was lower with landiolol {(JPY 78,956 (EUR 493), JPY 82,232 (EUR 514)}, suggesting potential savings of millions in healthcare expenditure. Sensitivity analyses supported the robustness of these findings. Although adverse effects were not included in the model, the study concluded that landiolol represents a cost-saving and diagnostically efficient option in this clinical setting [42].

Moreover, landiolol has shown potential as a cardioselective tracer for myocardial perfusion imaging. In a study, it was successfully radiolabeled with [^131^I] using chloramine-T, achieving a high radiochemical yield of 98% and demonstrating stability for up to 48 h. Purity of the compound was verified through thin-layer chromatography, electrophoresis, and high-performance liquid chromatography. Biodistribution studies revealed substantial heart uptake (45.0 ± 0.19% ID/g at 2 min post-injection), with a favorable heart-to-blood ratio maintained within 60 min, supporting its potential as a novel imaging agent for cardiac applications [43].

While the clinical utility of landiolol is supported by growing evidence, several limitations warrant discussion. Much of the existing data derive from small-scale or region-specific studies, particularly in East Asia, which may limit the extrapolation of results to broader, more diverse populations. Multicenter, randomized trials involving Western populations are necessary to validate efficacy, safety, and cost-effectiveness across healthcare settings. Moreover, long-term outcomes associated with landiolol use—such as its impact on mortality, recurrent ACS events, and chronic heart failure progression—remain to be fully established.

Integration of landiolol into ACS and imaging protocols appears promising based on current evidence, given its favorable pharmacological profile and clinical effectiveness. Its established role in the perioperative management of arrhythmias demonstrates its clinical utility in high-risk settings, suggesting that a similar expansion into broader CAD management may be both feasible and beneficial.

## Figures and Tables

**Table 1:** Summary of landiolol properties [6].

*Properties*	Landiolol
*Beta-1 Selectivity*	Very High
*Onset of action*	Rapid
*Half-Life*	Ultra-short (approx. 4 min)
*Duration of Action*	Very Short (stops quickly after infusion stop)
*Metabolism*	Plasma esterases
*Titration and Control*	Precise titration
*Effect on Blood Pressure*	Minimal BP lowering compared to other BBs
*Use in Acute Settings*	Ideal for acute rate control in ICU
*Common Indications*	Rapid rate control in AF, SVT, perioperative tachycardia
*Contraindications*	Severe bradycardia, advanced AV block, acute decompensated heart failure, severe hypotension
*Side Effects*	Hypotension, bradycardia, dizziness, potential worsening of heart failure
*Dosage*	Loading dose: 0.1 mg/kg IV bolus, followed by continuous infusion 5–40 mcg/kg/min

**Table 2:** Summary findings of landiolol in acute coronary syndromes.

Study ID	Type of Study	Population	Total Number (n), Landiolol Dosage/Comparator	Main Outcomes and Adverse Events
Miyamoto et al., 2020 [8]	Multi center RCT	STEMI	47 patients randomized to Landiolol (n = 23) Controls (n = 24)	🟂 MSI in the landiolol group was significantly reduced compared to control group (*p* < 0.001). 🟂 No significant differences in BP at recruitment, PCI and safety (composite endpoint of death, malignant ventricular arrhythmia, cardiogenic shock and atrioventricular block—AVB- at 24 h).
Hanada et al., 2012 [12]	Single center RCT	STEMI	96 patients after PCI randomized to Landiolol (n = 47) Controls (n = 49)	🟂 In the landiolol group LVEF increased from the acute to the chronic phase. 🟂 LVESV index did not change in either group. 🟂 LVEDV index increased in the control group only from the acute to the chronic phase. 🟂 HR decreased only in the landiolol group at 2 h (1 bradycardia). 🟂 Landiolol: 80.0 ± 2.3 bpm to 70.1 ± 1.7 bpm (*p* < 0.01). 🟂 Controls: (79.1 ± 1.7 to 77.0 ± 1.8 bpm, *p* = NS). 🟂 No other difference of cardiovascular events, SDP or DBP.
Fujita et al., 2010 [16]	Single center RCT	AMI	48 patients randomly assigned to Landiolol (n = 25) Controls (n = 23)	🟂 LVEF was better in the landiolol group but not statistically significant. 🟂 In 6 months, BNP was significantly lower in the landiolol group. Infarct size was comparable.
Fujita et al., 2012 [17]	Single center RCT	AMI	79 patients randomly assigned to Landiolol (n = 42) Controls (n = 37)	🟂 LVDV and LVEF improvement was better in landiolol group both by UCG and TcMIBI scintigraphy and statistically significant. 🟂 LVSV and BNP were not different.
Higuchi et al., 2010 [13]	Single center RCT	Anterior AMI	26 patients after PCI Landiolol (n = 14) Controls (n = 12)	🟂 Peak-CK and CK-AUC were significantly lower in landiolol group (*p* = 0.0394). 🟂 One event of bradycardia. No other adverse cardiac events.
Kiyokuni et al., 2016 [11]	Single-center Observational	NSTEMI STEMI	115 patients Landiolol (n = 55) Controls (n= 60)	🟂 Higher rate of an STR and MBG. Landiolol use was an independent predictor of an STR (OR: 2.99, 95% CI 1.25–7.16, *p* = 0.014). 🟂 Incidence of NSVT (*p* = 0.014), hypotension (*p* = 0.046) and progression of Killip class (*p* = 0.028) were lower in the landiolol group. 🟂 In 12 m follow-up, HF requiring hospitalization was lower in the landiolol group. Cardiac death, non-fatal MI/stroke and target vessel revascularization were similar among groups.
Hoshi et al., 2012 [10]	Single center Observational	ACS	Landiolol (n = 22)	🟂 HR reduction was significant (from 87 ± 11 to 72 ± 8 beats/min, *p* < 0.001). Two events of bradycardia. 🟂 No difference in SBP and DBP
Sun et al., 2023 [7]	Meta-analysis 7 RCTs	STEMI	3 Landiolol studies vs. placebo Hanada et al., 2012 [12] Kiyokuni et al., 2016 [11] Miyamoto et al., 2021 [9]	🟂 VT/VF: no significant difference (*p* = 0.34). 🟂 Bradycardia/AVB: no significant difference (*p* = 0.89). 🟂 Cardiogenic Shock: no significant difference (*p* = 0.98). 🟂 =HF readmission: no significant difference (*p* = 0.16).

**Table 3:** Summary findings of landiolol in computed tomography coronary angiography.

Study ID	Type of Study	Study Population (Main Characteristics)	Total n, Landiolol Dosage/Comparator	Main Outcomes and Adverse Events
Jinzaki et al., 2013 [20]	Multi- Center RCT	Adults with suspected CAD Inclusion: chest pain with positive findings on exercise (ECG) or positive findings on MBF/cardiac US	n = 183 patients Permuted-block randomization 3 groups n1 = 58: 0.06 mg/kg of landiolol n2 = 61: 0.125 mg/kg of landiolol n3 = 64: placebo All patients received 300–600 mg of nitroglycerin first	(Efficacy) HR at the time of CTCA: lower mean HR values in the landiolol groups (and, respectively) lower rates in the high-dose group (*p* = 0.002). n1 = 78.9 ± 9.2 to 67.6 ± 8.7 bpm (*p* = 0.003) n2 = 79.4 ± 9.6 to 62.6 ± 7.8 bpm (*p* < 0.001) n3 = 77.6 ± 10.0 to 73.7 ± 11.8 bpm (*p* = NS) HR reduction was significantly greater in both landiolol groups (both *p* < 0.001), and in the 0.125 mg/kg group, the achieved rate was lower (*p* = 0.007). The rapid reduction started immediately, became significant at 15 min and stopped being significantly lower than the placebo group after 30 min in both landiolol groups. (Safety) BP: no difference after 30 min. Adverse events: no difference among groups. CTCA analysis: Per patient and per artery analysis: correct classification proportion significantly higher in the 0.125 mg/kg group. Per segment analysis: assessable segments and correct classification proportion significantly higher in both landiolol groups.
Hirano et al., 2014 [21]	Multi Center RCT	Patients ≥ 20 years with suspected CAD Inclusion: (1) presented with stable angina (2) HR 70–90 bpm before nitrates	n = 258 patients Permuted-block randomization 2 Groups n1 = 130: 0.125 mg/k of landiolol n2 = 128: placebo group Bolus injection of study drug CCTA after 4–7 min.	(Efficacy) HR: significantly lower in the landiolol group (62.6 ± 8.5 bpm vs. 72.9 ± 12 bpm, *p* < 0.0001). HR reduction was significantly higher in the landiolol group (−19.1 ± 8.1% vs. −5.9 ± 9.7%, *p* < 0.0001). No significant difference was any longer found at 30 min after administration. (Safety) BP: mean SBP significantly lower in landiolol group (125.1 ± 20.7 mmHg vs. 132.7 ± 20.7 mmHg, *p* < 0.05), but recovered to the baseline value at 30 min. No serious adverse event or event requiring treatment. CTCA analysis: Image Quality Score: both at optimal and at mid-diastole reconstruction, a score of 2 or 3 was significantly higher in the landiolol group per subject, vessels and segment (*p* < 0.0001).
Nakamura et al., 2014 [28]	Single Center RCT	Patients who underwent CTCA	n = 354 patients 3 groups n1 = 188 (bolus dose of 0.125 mg/kg) n2 = 213 (bolus dose + 3.75 mg) n3 = 277 oral propranolol 1.5 h before CT	(Efficacy) HR was significantly lower in the propranolol group (61.6 ± 8.0 bpm) than in the n1 group (64.1 ± 7.4/min, *p* < 0.001), but there was no significant difference in the image quality (*p* = 0.91). Average HR tended to be lower in the n2 group (67.2 ± 6.9/min) compared with the n1 group (69.0 ± 6.9/min, *p* = 0.10), and there was a significant difference in image quality between these two groups (*p* = 0.02). (Safety) A patient developed bradycardia and another hypotension. They were asymptomatic, and both recovered after 5 min and 10 min, respectively.
Hirano et al., 2013 [22]	Multi Center Observational	Patients with suspected ischemic cardiac disease	n = 90 patients 3 groups of landiolol n1 = 0.125 mg/kg (Group L) n2 = 0.25 mg/kg (Group M) n3 = 0.5 mg/kg (Group H) CCTA 3–7 min after administration	(Efficacy) HR reduced in all groups in a dose-dependent way (15.55 ± 6.56% in Group L, 16.48 ± 7.80% in Group M, and 21.49 ± 6.13% in Group H). (Group L vs. Group H, *p* = 0.0008; Group M vs. Group H, *p* = 0.0109). (Safety) BP decrease was minimal in all groups and returned to baseline levels after administration. CTCA analysis: coronary stenosis was diagnosable in all groups with no significant difference.
Isobe et al., 2008 [23]	Prospective Observational	Patients with known or suspected CAD undergoing MSCT on admission to hospital	n = 145 patients receiving landiolol continuously injected 15 min before starting MSCT CAG and stopped immediately after. Final dose of landiolol hydrochloride was 0.036 ± 0.005 mg·kg^−1^·min^−1^	(Efficacy) HR: significantly reduced during injection of landiolol hydrochloride (51.8 ± 3.1 bpm, *p* < 0.0001), quickly recovered 15 min after cessation of injection (62.8 ± 7.9 bpm) and was maintained until sleep. All patients achieved the target HR ≤ 55 bpm at the start of the CT scan. Mean time to reach the target HR was 13.4 ± 3.8 min. HR variability was significantly reduced during CT acquisition compared with before the administration of landiolol. (Safety) BP: no significant changes were observed. No adverse effects were reported. (CCTA analysis) Per-segment analysis and per-artery analysis were performed.
Koyoshi et al., 2018 [29]	Single Center Prospective	CTA for suspected CAD or at least one cardiac risk factor	n = 176 patients One bolus injection of landiolol (16.1 ± 7.4 mg) 4 min before scan	(Efficacy) HR before administration, after administration and at the end of scan: 83 ± 10 bpm, 62 ± 7 bpm and 69 ± 8 bpm, respectively (*p* < 0.001)) HR upon entry to the CT room: 70–79 bpm (74 ± 3 bpm) (n = 76); 61 ± 6 bpm during scan. 80–89 bpm (84 ± 3) (n = 60); 63 ± 7 bpm during scan. ≥90 bpm (98 ± 6) (n = 40); 65 ± 7 bpm during scan. (Safety) SBP/DBP from 136 ± 17/80 ± 12 before scan to 123 ± 18/72 ± 12 mmHg after scan; eight patients (4.5%) had adverse events, but none was severe or required stopping drug administration.
Kido et al., 2016 [24]	Multi Center Prospective	CTCA for suspected ischemic heart disease	n = 219 patients	(Efficacy) Mean HR after administration was 59.9 ± 6.4 bpm compared to 69.3 ± 7.3 bpm before; *p* < 0.001. 80% of the patients achieved HR ≤ 65 bpm. (Safety) HR and BP of all the patients recovered after the scan. No adverse events during the study. (CCTA analysis) The mean radiation dose was 50%, derived from the inferred dose before use of landiolol (4.5 ± 3.2 vs. 9.0 ± 3.7 mSv; *p* < 0.001).
Kokubo et al., 2022 [25]	Single Center Retrospective	CTCA for suspected ischemic heart disease	n = 142/244 patients received landiolol	(Efficacy) HR decreased significantly (from 66.2 ± 9.74 to 53.4 ± 7.2 bpm, *p* < 0.001). No significant changes in EF.
Barwig et al., 2025 [26]	Single Center Retrospective	Patients with HR > 60 bpm underwent CTCA	n = 37 patients n1: 23 patients without oral BB premedication n2: 14 with prior BB use Fractional administration 1–5 doses (60 mg) Mean dose (± SD): 0.526 ± 0.3 mg/kg	(Efficacy) HR ≤ 60 bpm was achieved in 13 patients (35%), and a HR ≤ 65 bpm was achieved in 25 patients (68%). Mean ± SD of HR before and during CT: −11 ± 9 bpm in total, −14 ± 10 bpm in group 1 and −6 ± 5 bpm in group 2. Statistically significant in all groups. (Safety) No adverse effects occurred.
Osawa et al., 2013 [27]	Single Center Observational	Patients who underwent MDCT coronary angiography	n = 66 patients received landiolol	(Efficacy) HR significantly reduced 5 min after injection of landiolol and recovered shortly after. (Safety) SBP did not decrease significantly. Adverse events not observed in patients receiving landiolol.

## Abbreviations

The following abbreviations are used in this manuscript:

CAD	Coronary artery disease
BBs	Beta Blockers
pPCI	primary Percutaneous Coronary Intervention
bpm	beats per minute (bpm
CTCA	Computed Tomography Coronary Angiography
ACSs	Acute coronary syndromes
ESC	European Society of Cardiology
STEMI	ST-elevation myocardial infarction
NSTEMI	Non-ST-elevation myocardial infarction
SVTs	Supraventricular tachyarrhythmias
AF	Atrial Fibrillation
HR	Heart Rate
MSI	Reduction in Myocardial Salvage Index
CMR	Cardiac Magnetic Resonance
RCTs	Randomized Control Trials
STR	ST-segment Resolution
MBG	Myocardial Blush Grade
VF	Ventricular Fibrillation
VT	Ventricular Tachycardia
NSVT	Non-sustained Ventricular Tachycardia
BNP	B-type Natriuretic Peptide
LVEF	Left Ventricular Ejection Fraction
LVEDV	Left Ventricular End-Diastolic Volume
LVEDV	Left Ventricular End-Systolic Volume
SBP	Systolic Blood Pressure
DBP	Diastolic Blood Pressure
AVB	Atrioventricular Block
CK	Creatine Kinase
CK-AUC	CK area under the curve
EF	Ejection Fraction
LVWSI	Left Ventricular Wall Motion Score Index
APN	Adiponectin
IRI	Ischemia-Reperfusion Injury
IPC	Ischemic Precondition
FEV1	Forced Expiratory Volume in 1 s
